# hmmibd-rs: An enhanced hmmIBD implementation for parallelizable identity-by-descent detection from large-scale Plasmodium genomic data

**DOI:** 10.21203/rs.3.rs-7004070/v1

**Published:** 2025-07-02

**Authors:** Bing Guo, Stephen F. Schaffner, Aimee R. Taylor, Timothy D. O’Connor, Shannon Takala-Harrison

**Affiliations:** University of Maryland School of Medicine; Broad Institute of MIT and Harvard; Institut Pasteur, Université Paris Cité; University of Maryland School of Medicine; University of Maryland School of Medicine

**Keywords:** Identity-By-Descent, Hidden Markov Model, Parallelization, Population Genomics, Recombination Rate Map, Plasmodium

## Abstract

**Background:**

Identity-by-descent (IBD), which describes recent genetic co-ancestry between pairs of genomes, is a fundamental concept in population genomics. It has been used to estimate genetic relatedness, detect selection signals, and understand population demography. The IBD detection method *hmmIBD* demonstrates high accuracy in inferring IBD segments between haploid genomes, including *Plasmodium falciparum*, and is widely used in malaria genomic surveillance. However, the current single-threaded implementation of *hmmIBD* does not utilize the full capacity of multi-processor computers, making it difficult to apply to large data sets, and does not accommodate non-uniform recombination rates across the genome.

**Methods:**

We developed an enhanced implementation of *hmmIBD* in the Rust programming language, named *hmmibd-rs*, which leverages multi-threaded computing to parallelize IBD inference over genome pairs and which supports optional, user-defined recombination rate maps for more accurate IBD detection and filtration from genomes with non-uniform recombination. We further streamlined large-scale IBD detection by incorporating auxiliary built-in functionalities to preprocess input directly from the standard binary variant call format (BCF) and filter IBD output to reduce disk usage.

**Results:**

Our new implementation significantly reduces IBD detection computation time nearly linearly with the increased number of CPU threads used; using 128 threads shortens IBD detection time from 5.2 days to 1.3 hours for 220 million pairs of simulated *Plasmodium falciparum*-like chromosomes, increasing computational speed by approximately 100x over the single-threaded *hmmIBD* algorithm. Incorporating non-uniform recombination rates in *hmmibd-rs* enhances the accuracy of IBD inference by mitigating the overestimation of IBD breakpoints in recombination cold spots and their underestimation in hot spots. It also improves IBD segment length filtration, reducing the false positive rate in recombination cold spots and the false negative rate in hot spots. When applied to empirical data sets, *hmmibd-rs* completes the detection of IBD from MalariaGEN Pf7 (n ≈ 10,000 monoclonal samples) within hours, enabling a single-day IBD analysis pipeline for large genomic data sets.

**Conclusion:**

*hmmibd-rs* builds upon, accelerates, and enhances *hmmIBD* for efficient and accurate IBD detection, serving as a crucial tool for advancing large-scale malaria genomic surveillance.

## BACKGROUND

Identity-by-descent (IBD) refers to alleles or genomic regions (segments) that are identical between two individuals/genomes due to shared ancestry. For species with high recombination rates relative to mutation rates, such as malaria parasites, metrics that leverage recombination, e.g., IBD, capture finer-scale and higher-resolution dynamics in population demography [1–3]. IBD, inferred from malaria parasite genomic data, conveys important information about the recent history of populations, including genetic relatedness within and between populations, loci under natural selection, and time-specific demography (effective population size and population structure), thus playing a crucial role in malaria genomic surveillance [4–14].

Accurate detection of IBD segments often requires genotype data with sufficient marker density [4, 15, 16]. Species with a high ratio of recombination rate to mutation rate, such as the malaria parasite *Plasmodium falciparum*, tend to have a (common variant) marker density two orders of magnitude lower than that of humans [9, 15, 17–20]. Thus, an IBD detection algorithm robust to low marker density is crucial for high-recombining species. Our accompanying project suggests that *hmmIBD* stands out among many other IBD detection methods, uniquely providing high-quality IBD segment calls, including shorter segments, that allow for the generation of accurate results even for quality-sensitive inferences [16]. Despite its high accuracy and wide adoption in malaria research, *hmmIBD* could be improved in several areas: it uses only a single thread of multi-core CPUs, it assumes a uniform recombination rate across the genome, and it requires substantial preprocessing of input data into a specific format prior to analysis [5]. These limitations may prevent its wider application to larger data sets or to analyses that rely on a non-uniform genetic map [20, 21], an important consideration given the demand for analysis of large-scale whole-genome sequence (WGS) data [20] and opportunities to construct high-resolution recombination rate maps based on recent genetic crosses [22, 23].

In this work, we addressed these limitations by reimplementing and enhancing the Hidden Markov Model described in the original paper [5] using the Rust programming language. Our new implementation, *hmmibd-rs*, offers three key features: parallelized IBD inference, support for non-uniform recombination rates, and streamlined data management.

## METHODS

We enabled parallelization for the HMM inference process at the level of single haploid genome pairs or groups of haploid genome pairs. In our reimplementation, we first modularized the original algorithm into multiple components, including data processing modules and different subcomponents of the HMM inference process. Relying on the modular structure, we then isolated the HMM inference process for a genome pair as the basic unit for parallelization. The original sequential HMM inference process, iterated over genome pairs, was converted into parallelizable tasks, utilizing the *Rayon* crate, a library designed for data parallelism [24]. In addition, we provide options to optimize memory usage based on parameters such as the maximum number of alternative alleles per locus and the output file buffer sizes.

We enabled non-uniform recombination rates for HMM inferences and IBD segment filtration using user-provided genetic maps. For HMM inference, we updated the term $e^{-kp\;d_{t}}$ in the transition probabilities matrix [5] to the term $e^{-kc_{t}}$, where ρ is the recombination rate per generation per bp, and *d*_*t*_ is the physical distance between the *t* th marker and the *t*−1 th marker, and *c*_*t*_ is the genetic distance between the two markers given by the user. As *c*_*t*_ = *ρ d*_*t*_, our new implementation maps physical distances between markers to genetic distance, and directly uses genetic distance *c*_*t*_ for HMM inference, thus removing the assumption of a uniform recombination rate along the genome. Besides the HMM inference steps, we also allow the usage of a recombination rate map for post-HMM inference, built-in IBD segment length filtration in genetic units, which is usually needed for analyses based on IBD segments since short IBD segment estimates are more error-prone and often filtered out before downstream analyses [15, 16, 25].

We improved the efficiency and ergonomics of input and output data management. We developed a simple, cross-platform auxiliary library *bcf_reader* in Rust and used it to process input data directly from the common genotype file format, binary variant call format (BCF). Based on this library, we implemented two main built-in functions. The first main function is to construct haploid genomes by replacing heteroallelic genotype calls in monoclonal samples with dominant alleles if the total allele depths (via the command line option *min_depth*) and the fraction of reads supporting the dominant alleles (via the options *min_ratio* and *min_r1_r2*) are high; otherwise, these are set to missing (as detailed in Supplementary Table 1). We note that the default criteria for determining whether to use dominant alleles or missing data are somewhat subjective: users may opt for stringent thresholds, such as setting all heteroallelic calls to missing data, which comes with the caveat of removing more sites and samples during the subsequent genotype filtering step, or more permissive ones to include all heteroallelic calls by using the allele with the highest read support, which may introduce substantial genotyping errors. The second built-in function is to iteratively filter samples and sites (based on the missingness of genotype calls) to obtain high-quality genotype data while retaining balanced numbers of markers and samples (Supplementary Table 1). The dominant-allele-based haploid genome construction from BCF files is a heuristic strategy for working with monoclonal samples. Haploid genomes from polyclonal infections may be inferred via external deconvolution programs like *DEPloid* or *DEPloidIBD* [26, 27] and provided to *hmmibd-rs* in a traditional table format used in *hmmIBD*.

We have included additional options, made available via the command line interface, to customize HMM parameters and data management parameters (e.g.,BCF processing, IBD filtering, output buffering, and optional suppressing) to allow the user to balance analytical needs and computational/storage efficiency. Additionally, *hmmibd-rs* is designed to be fully compatible with *hmmIBD* to facilitate the transition to *hmmibd-rs*, and, by default, generates both files for IBD segments and files for the fraction of sites IBD (estimates of genetic relatedness) like *hmmIBD*.

Methods used for simulation, measurement of computation time, and downstream analysis of detected IBD segments are similar to our previous analysis [14, 16], with further description provided in the Supplementary Methods. Details of these analyses can be found in the related pipeline and source code listed in the Availability of Data and Materials.

## RESULTS

**Figure 1:**
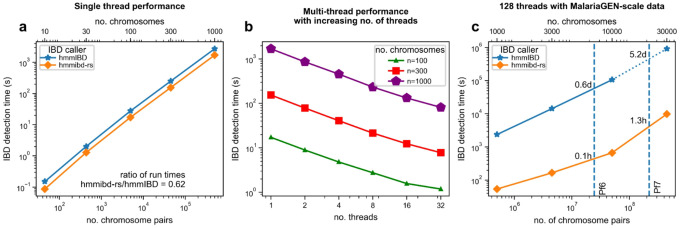
*hmmibd-rs* significantly reduces the computation time for IBD detection in simulated *P. falciparum*-like chromosomes when compared to *hmmIBD*. a, Comparison of single-thread runtime of *hmmibd*-*rs* and *hmmIBD* for simulated data with different sample sizes. b, Multithreading performance of *hmmibd-rs*. c, Runtime for detecting IBD from simulated data on a MalariaGEN-scale using *hmmibd-rs* with 128 threads. Note that in (a and c), sample sizes are indicated as both the number of chromosomes (top horizontal axis ticks) and the number of chromosome pairs (bottom horizontal axis ticks). Dotted line in (c) indicates that the top right data point (star) is an estimate based on the extrapolation of data points involving smaller sample sizes.

Our new implementation, *hmmibd-rs*, improves the computational efficiency of HMM IBD inference both by increasing single-thread performance and by enabling multithreading. When both *hmmibd-rs* and *hmmIBD* are forced to use a single thread, run times with *hmmibd-rs* were about 40% shorter than those of *hmmIBD* (Fig. 1a), due to the more compact memory representation of the genotype matrix and reduced disk read and write operations in *hmmibd-rs*. When multithreading is enabled, the performance of *hmmibd-rs* is almost linear with respect to the number of threads and the number of genome pairs. To test the performance on a large data set, we ran *hmmIBD* and *hmmibd-rs* on simulated *P. falciparum*-like genomic data with a sample size of up to n = 30,000, which is the same order of magnitude as the MalariaGEN Pf7 data set (n > 21,000). Our new implementation completed the IBD detection from the simulated data set in 1.3 hours using 128 threads with the AMD EPYC 9654 CPU model, whereas the single-threaded *hmmIBD* took an estimated 5.2 days to complete IBD detection (Fig. 1c). Additionally, when IBD segment length filtering options were used, the resulting file sizes were largely reduced (Supplementary Table 2). Thus, this new implementation, in this example, can accelerate the process by two orders of magnitude.

**Figure 2:**
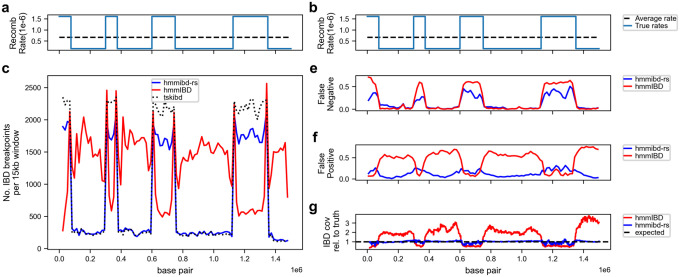
*hmmibd-rs* improves the accuracy of reported IBD segments by incorporating a non-uniform recombination rate map in the HMM algorithm and the IBD length filtering step. a-b, the true, non-uniform recombination rate map (blue line) and the constant chromosome-wide average rates (black dotted line). c. Number of detected IBD breakpoints (two ends of IBD segments over 2 centimorgans, cM) in 15kb windows along the simulated *P. falciparum*-like genomes. e-f, False negative rate (e) and false positive rate (f) of detected IBD segments (over 2 cM) using IBD segment overlapping analysis (see Methods). g, Coverage of detected IBD segments (over 2 cM), normalized to the coverage of true IBD segments (over 2 cM, determined by *tskibd* [14]). Note: 1) The true (non-uniform) rate was used to simulate the genotype data; 2) *tskibd* is used to generate true IBD segments from simulated genealogy trees, and the true recombination rate is used for true IBD length calculation and filtration; 3) For *hmmibd-rs*, the true rate was used for both the HMM inference and the IBD length filtration; 4) For *hmmIBD*, the average rate is used for both the HMM and length filtration steps. See Supplementary Figure 1 for similar analyses using the true rate for length calculation and filtering for *hmmIBD*-inferred IBD segments. Also, see Supplementary Figure 1 for the IBD breakpoint analysis when all IBD segments are included (without length filtering).

To understand how recombination rate misspecification affects IBD detection, we simulated genomes with a non-uniform recombination rate map that had the same mean recombination rate as the P. falciparum genome (Fig. 2a and b). When focusing on IBD segments ≥ 2 cM and using the average recombination rate (*hmmIBD*), we found that the number of ends (breakpoints) of the detected IBD segments decreases for recombination hot spots and increases for cold spots when compared to those using the true non-uniform rates (*hmmibd-rs*) (Fig. 2c). Consistently, the error rates (false negative rates and false positive rates, Fig. 2e and f) and deviation from the true IBD coverage pattern (Fig. 2g) were significantly higher in *hmmIBD* results than in *hmmibd-rs*. The differences between *hmmIBD*- and *hmmibd-rs*-derived IBD segments are largely reduced when using the true rates to calculate length used to filter segments inferred by *hmmIBD*, suggesting that an accurate recombination map is important for filtering IBD segments by length in genetic units (Supplementary Fig. 1). To test whether the recombination rate affects HMM inference, we analyzed unfiltered IBD segments when called with true (*hmmibd-rs*) and average rates (*hmmIBD*). We showed that rate misspecification indeed affects the detection of IBD breakpoints (Supplementary Fig. 2). The general underestimation of IBD breakpoints in recombination hotspots likely arises from two main factors: the IBD-merging bias in the HMM and the high error rates caused by low marker densities per genetic unit. In addition, this issue is aggravated by reduced state switching rate in the HMM and aggressive IBD removal in length filtering due to recombination rate misspecification (see Supplementary Note for more details). This finding highlights the importance of accurately characterizing the local recombination rate variation and using it to improve the detection and filtration of IBD segments.

**Figure 3:**
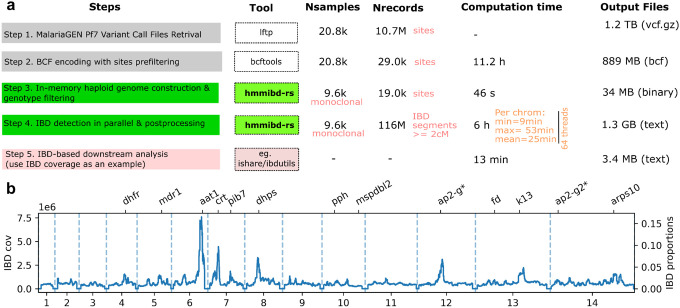
The *hmmibd-rs*-based, VCF-to-IBD pipeline enables fast and streamlined detection of IBD segments from the MalariaGEN Pf7 data set within a single day. a, Descriptions, tools involved, numbers of samples and records analyzed, computation time, and output file sizes over the five steps of the VCF-to-IBD pipeline. b, Example analysis (IBD coverage) of the detection of IBD segments from the MalariaGEN Pf7. Genes of interest (potentially under positive selection) were labeled above the plot.

Another obstacle in the *hmmIBD*-based analytical pipeline is the need for an *hmmIBD*-specific format for the input data, which adds a data formatting step to IBD detection and downstream analysis, which is particularly cumbersome if iterative filtering of samples and variants is done. We mitigated these issues by implementing optional, built-in, all-in-memory functions for iterative sample and site filtering, and for haploid genome construction using dominant alleles (Supplementary Table 1). We presented a simple pipeline based on these features, implemented in *hmmibd-rs*, to demonstrate its applicability to large-scale WGS data sets, from VCF/BCF files to IBD-based estimates, which includes genotype filtering and haploid genome construction (*bcftools* [28] and *hmmibd-rs*), IBD calling (*hmmibd-rs*), and IBD coverage calculation. We were able to finish IBD calling from the raw genotype call files within a single day using 64 threads CPU (Fig. 3a), with the majority of time spent on *bcftools* for the initial genotype filtering step. As a proof of data show signals of positive selection (Fig. 3b) consistent with previous reports [11, 14].

## DISCUSSION

This study presents an improved implementation of *hmmIBD* with three important features for large-scale population genomics: high computational performance, optional recombination rate map specification, and improved data management. Compared to other probabilistic IBD (segment) detection methods popular in malaria research, including *hmmIBD* [5], isoRelate [6] and DEploidIBD [27], *hmmibd*-*rs* is the first attempt to leverage the memory-safe language Rust and its rich ecosystem to embrace the era of large-scale genomics by enabling computational parallelization, employing a standard input format and lowering difficulty of long-term software maintainability and further development to incorporate more complex models. Although *hmmibd-rs* has mainly been applied to *Plasmodium* data, it is expected to work with data from other sexually recombining species with high recombination rates for which haploid genomes can be constructed [29], which may include non-*Plasmodium* Apicomplexan species, such as *Theileria parva* [30], and insects such as *Apis mellifera* [31, 32] and fungi such as Saccharomyces cerevisiae [33, 34]. The new features of *hmmibd-rs* including its parallelizability and support of a non-uniform genetic map may allow the detection of inter-individual IBD segments from phased data on diploids, e.g. mosquitoes, by treating each phase of a diploid individual as a haploid genome. However, given advances in human genetics, a superior approach for diploids may also exist.

One caveat of our analyses of *hmmibd-rs* is the lack of reliable non-uniform recombination rates for empirical data sets of high-recombining species like *P. falciparum*, despite initial efforts to estimate either the average rate or high-resolution rate maps based on limited samples [22, 23]. Ongoing work is needed to estimate high-resolution recombination rate maps based on existing genetic cross data, as well as WGS data from large-scale population samples [21, 22, 35–37]. This will allow further evaluation of biases in IBD-based analysis due to the use of a simple average rate in empirical data. We also note that the optional built-in function that constructs haploid genomes based on dominant alleles is misspecified for polyclonal samples. Using more advanced genotype deconvolution tools, such as *DEploid* and *DEploidIBD* [26, 27], may better utilize polyclonal infections, although it may significantly increase the computational burden.

## CONCLUSION

*hmmibd-rs* enhances the original IBD detection algorithm *hmmIBD* with key features that significantly accelerate IBD detection from large-scale genomic data and enable the incorporation of a genetic map for improved accuracy in genomes with non-uniform recombination. The new implementation allows for more efficient, accurate, and streamlined IBD-based analysis of *Plasmodium* genomes, which will contribute to the timely malaria genomic surveillance.

## Supplementary Material

Supplementary Files

This is a list of supplementary files associated with this preprint. Click to download.

- hmmibdrsmanuscriptsuppl.docx

## Data Availability

Source code for *hmmibd-rs* in Rust is available at https://github.com/bguo068/*hmmibd-rs*; this repository also includes a version of *hmmIBD* in C, modified to allow users to specify the average recombination rate via a command line option. Source code for the cross-platform library for reading BCF file format, bcf-reader is available at https://github.com/bguo068/bcf-reader. The pipeline that simulates data and benchmarks and characterizes *hmmibd-rs* and *hmmIBD* is accessible at https://github.com/bguo068/hmmibd-rs-bench. The pipeline for a full demonstration of *hmmibd-rs’s* application to MalariaGEN Pf7 is provided at https://github.com/bguo068/hmmibd-rs-bench-empirical. The genotype data of the MalariaGEN Pf7 data set [20] and its sample meta information, including *F*_*ws*_ estimates, are publicly available at https://www.malariagen.net/resource/34/.
